# Antiviral Effect of Lithium Chloride and Diammonium Glycyrrhizinate on Porcine Deltacoronavirus In Vitro

**DOI:** 10.3390/pathogens8030144

**Published:** 2019-09-09

**Authors:** Xiaofeng Zhai, Shilei Wang, Mengyan Zhu, Wei He, Zhongzhou Pan, Shuo Su

**Affiliations:** MOE International Joint Collaborative Research Laboratory for Animal Health & Food Safety, Jiangsu Engineering Laboratory of Animal Immunology, Institute of Immunology, College of Veterinary Medicine, Nanjing Agricultural University, Nanjing 210095, China (X.Z.) (S.W.) (M.Z.) (W.H.) (Z.P.)

**Keywords:** PDCoV, lithium chloride, diammonium glycyrrhizinate, apoptosis

## Abstract

Porcine deltacoronavirus (PDCoV) is an emerging global swine virus that has a propensity for interspecies transmission. It was identified in Hong Kong in 2012. Given that neither specific antiviral drugs nor vaccines are available for newly emerging porcine deltacoronavirus, searching for effective antiviral drugs is a high priority. In this study, lithium chloride (LiCl) and diammonium glycyrrhizinate (DG), which are host-acting antivirals (HAAs), were tested against PDCoV. We found that LiCl and DG inhibited PDCoV replication in LLC-PK1 cells in a dose-dependent manner. The antiviral effects of LiCl and DG occurred at the early stage of PDCoV replication, and DG also inhibited virus attachment to the cells. Moreover, both drugs inhibited PDCoV-induced apoptosis in LLC-PK1 cells. This study suggests LiCl and DG as new drugs for the treatment of PDCoV infection.

## 1. Introduction

PDCoV, first discovered in Hong Kong in 2012, broke out in the United States in early 2014 and then spread to China, Thailand, and Canada. As a novel coronavirus, PDCoV can cause severe diarrhea and vomiting, with high mortality in piglets, which can lead to serious economic loss in the swine industry [1,2,3]. Coronaviruses (CoVs) are enveloped positive-stranded RNA viruses that are classified into four genera including *Alpha*, *Beta*, *Gamma*, and *Deltacoronavirus*. CoVs are prone to interspecies transmission [4]. In particular, *Betacoronaviruses* include SARS-CoV and MERS-CoV, which can cause lethal respiratory infections in humans and are notable examples of interspecies transmission [5,6]. Cross-species transmission, facilitated by the widespread prevalence of CoV in mammals and birds, and genetic diversity, driven by high mutation rates and high frequency of recombination, increase the chance of successful adaptation to a new host [7].

PDCoV, belonging to the genus *Deltacoronavirus* and the family Coronaviridae within the order Nidovirales, is a recently discovered CoV of unknown origin, which has a wide host range, including wild birds, swine, and other mammals [8,9]. The PDCoV genome is approximately 25 kb and has seven open reading frames, i.e., open reading frame 1a/1b (ORF1a/1b), spike (S), envelope (E), membrane (M), nucleocapsid (N), NS6, and NS7. As an emerging swine enteropathogenic coronavirus, PDCoV has strong pathogenicity and can cause watery diarrhea, vomiting, and high mortality in piglets, posing a potential threat to the swine husbandry. The post-infection mortality of suckling piglets can reach 30% to 40%, causing serious economic losses to the pork industry [10]. PDCoV efficiently infects cells of several species, including swine, human, and chicken. Ectopic expression of porcine aminopeptidase N (APN) rendered non-susceptible cells susceptible to PDCoV infection and greatly enhance PDCoV infection in HeLa cells [11]. This suggests that PDCoV has the potential to spread between species and may be a threat to humans, as closely related viruses have led to outbreaks of deadly diseases such as SARS and MERS [12,13]. PDCoV global distribution in swine and its potential for transmission to multiple hosts are alarming. Pigs are the second largest livestock species and act as intermediate hosts for multiple zoonotic transmissions, emphasizing the need for studying the zoonotic potential of PDCoV and the development of antiviral drugs [14].

Given the lack of specific antiviral therapies to control PDCoV infection, searching for effective small-molecule inhibitors active against PDCoV is a high research priority. Antiviral reagents include directly acting antivirals (DAAs), which can directly act on viral components (proteins or genomes), and host-acting antivirals (HAAs), which can act on hosts [15,16,17]. Although DAAs are more virus-specific, viruses quickly develop resistance [18]. Antiviral methods based on host cellular mechanisms have potential advantages in reducing virus drug resistance and dealing with viral emergencies. Therefore, the search and development of HAAs has aroused more and more interest. An interesting feature of HAAs is their broad-spectrum antiviral activity, which allows for immediate treatment without a lead time being required to develop a specific therapy [19]. With the emergence of new viruses threatening human health, the discovery of new functions in existing clinical drugs is often used in the screening of modern emergency therapies. Traditional broad-spectrum antiviral drugs have shown antiviral activity against many types of viruses [17]. For example, ribavirin has antiviral activity against Hantavirus [20], H1N1 influenza virus [21], Dengue virus [22], and Chikungunya virus [23]. Therefore, the discovery of new antiviral functions for existing clinical drugs is still an important strategy against PDCoV infection. 

Here, the antiviral activities of LiCl and DG, which are both HAAs, were studied against PDCoV. LiCl can inhibit the replication of a variety of viruses, including Herpesvirus type 1 (HSV-1) [24], transmissible gastroenteritis virus (TGEV) [25], porcine reproductive and respiratory syndrome virus (PRRSV) [26], pseudorabies virus (PRV) [27], and porcine epidemic diarrhea virus (PEDV) [28]. LiCl can also regulate apoptosis and promote resumption of the synthesis of host proteins in infected cells [25,26]. Recent studies showed that LiCl could effectively inhibit the entry and replication of PEDV in Vero cells in a dose-dependent manner [28]. In addition, LiCl can inhibit early and late apoptosis induced by PEDV. PDCoV and PEDV belong to the family Coronaviridae and have similar genomic structure. However, antiviral drugs against PDCoV have not been researched. Therefore, we explored whether LiCl could inhibit infection by PDCoV. The licorice extract isolated from the rhizome of *Glycyrrhiza uralensis* has a strong antiviral activity [29]. In recent years, licorice extracts have been shown to have antiviral effects on many viruses including human immunodeficiency virus (HIV) [30], hepatitis B virus (HBV) [31], hepatitis A virus (HAV) [32], SARS-CoV [33], infectious bronchitis virus (IBV) [34], PRRSV [29], and PRV [27]. According to previous studies, DG pre-treatement inhibited PRV infection in a dose-dependent manner and reduced apoptosis [27]. Glycyrrhizin and DG have antiviral effect against SARS-CoV and IBV. Additionally, the water solubility of DG is better than that of glycyrrhizin, so we chose to verify the effect of DG on PDCoV. 

Here, we report that LiCl and DG inhibit the replication of the PDCoV strain SD2018/10 in LLC-PK1 cells in a dose-dependent manner. The antiviral activity of LiCl mainly occurs at the early stage of viral infection, while the antiviral activity of DG mainly occurs at the stage of viral attachment. In addition, both drugs inhibited PDCoV-induced apoptosis in LLC-PK1 cells. Therefore, the potential and theoretical bases of the two drugs as therapeutic drugs for PDCoV were validated.

## 2. Materials and Methods

### 2.1. Cells Culture and Reagents

LLC-PK1 cells (porcine kidney proximaltubular epithelial cells) (ATCC^®^ CL-101™) were grown in Dulbecco’s modified Eagle’s medium (DMEM, Hyclone, USA) supplemented with 10% fetal bovine serum (FBS, Gibco, Calsbad, USA) at 37 °C in a 5% CO_2_ incubator. LiCl (Sigma, St. Louis, MO, USA) and DG (TargetMol, Shanghai, China) were diluted in DMEM at 1 M and 10 mg/mL, respectively, and filter-sterilized.

### 2.2. Virus Stocks and Titration

The PDCoV strain SD2018/10 from our lab was used in this study. The number of infectious PDCoV particles was determined on the basis of the 50% tissue culture infectious dose (TCID_50_) in LLC-PK1 cells. Briefly, the cells were seeded into 96-cell plates and grown to 100% confluence for 24 h. Then, the cells were infected using 10-fold serial dilutions of virus samples. After incubation at 37 °C for 2 h, the cells were washed three times with PBS and incubated for 72 h. Titers are reported as TCID_50_ calculated by the method of Reed and Muench.

### 2.3. Cytotoxicity Assay

The cytotoxicity of LiCl and DG was assessed in vitro using the Cell Counting Kit-8 (CCK8, APE×BIO, Houston, TX, USA) according to the manufacturer’s instructions. LLC-PK1 cells were seeded into 96-cell plates and grown to 100% confluence for 24 h. After washing three times with PBS, the cells were treated with increasing concentrations of LiCl ranging from 0 to 100 mM or DG ranging from 50 to 5000 μg/mL. Mock-treated cells served as a control. After 36 h, the cells were washed with PBS and incubated with 90 μL DMEM and 10 μL of CCK-8 solution at 37 °C for 2 h. Absorbance was measured with a microplate reader (Tecan, M200 PRO, CH) at 450 nm. Cytotoxicity was expressed according to the following formula:

$$\mathrm{Cytotoxicity}(\%)\;=\;\frac{\left(\mathrm{Abs}\;\mathrm{sample})-(\mathrm{Abs}\;\mathrm{blank}\right)}{\left(\mathrm{Abs}\;\mathrm{negative}\;\mathrm{control})-(\mathrm{Abs}\;\mathrm{blank}\right)}\times 100$$

### 2.4. Antiviral Assay

To investigate the antiviral efficacy of LiCl and DG against PDCoV replication, cells were infected with PDCoV (MOI 0.05) at 37 °C for 1 h. Unbound viruses were removed by washing with cold PBS. The cells were then incubated with LiCl (60 mM) or DG (1250 μg/mL) at 37 °C for 24 h. As a control, another set of cells were infected with the same dose of PDCoV without drug treatment. Subsequently, the antiviral efficacy was evaluated by analysis of viral RNA (vRNA) levels and viral loads.

### 2.5. Analysis of the Effect of LiCl and DG on Viral Attachment

LLC-PK1 cells were seeded into 12-cell plates and grown to 100% confluence for 24 h. Non-toxic concentrations of LiCl (60 mM) or DG (1250 μg/mL) were mixed with the virus (MOI 0.05), and the mixtures were then added to the cells, followed by incubation for 1 h at 4 °C. As a control, the cells were infected with the same dose of PDCoV without drug treatment. After washing with cold PBS, the levels of vRNA in the cells were determined by real-time qPCR.

### 2.6. Analysis of the Viricidal Effect

PDCoV (MOI 0.05) was incubated for 2 h at room temperature or 37 °C in the absence or presence of LiCl (60 mM) or DG (1250 μg/mL) in a final volume of 1000 μL. After incubation, the infectious titer was determined by TCID_50_ in LLC-PK1 cells.

### 2.7. Analysis of the Effect of LiCl and DG on Viral Entry

The cells were infected with PDCoV (MOI 0.05) at 4 °C for 1 h. Unbound viruses were removed by washing with cold PBS. The cells were then incubated with LiCl (60 mM) or DG (1250 μg/mL) at 37 °C for 2 h. As a control, another set of cells were infected with the same dose of PDCoV without drug treatment. After removing the unbound viruses by washing with cold PBS (pH 3.0), the levels of vRNA in cells were determined by real-time qPCR.

### 2.8. Real-Time qPCR

Total cellular RNA was extracted using the RNApure Tissue & Cell Kit (CWBIO, Nanjing, China). The primers for PDCoV M and β-actin genes were: PDCoV-F, 5′-ACAATCGACCACATGGCTCCAA-3′, PDCoV-R, 5′-CAGCTCTTGCCCATGTAGCTTCA-3′, β-actin-F, 5′-CTCCATCATGAAGTGCGACGT-3′, β-actin-R, 5′-GTGATCTCCTTCTGCATCCTGTC-3′ [1]. The quantity of vRNA was determined using a LightCycler96 (Roche, Germany) and HiScript II One Step qRT-PCR SYBR Green Kit (Vazyme, Nanjing, China), according to the instructions of the manufacturer. The relative level of RNA expression was determined by the 2^−ΔΔCT^ method. β-actin mRNA level was used as a loading control. The mean RNA level of the mock-treated group was set at 100%.

### 2.9. Indirect Immunofluorescence Assay (IFA)

After infection, the cells were fixed with cold polysorbate for 30 min at room temperature and permeabilized with 0.2% Triton X-100 in PBS for 10 min. After washing five times with PBS, cthe ells were incubated with rabbit anti-N antibody (1:500) at 4 °C for 12 h. The cells were then incubated with FITC-conjugated goat anti-rabbit IgG (1:100, KPL, Colton, CA, USA) at 37 °C for 30 min. After washing five times with PBS, the cells were incubated with DAPI (1:1000, diluted in PBS) at room temperature for 20 min. Fluorescence was observed under a Nikon ECLIPSE TS100 fluorescence microscope (Nikon, Tokyo, Japan).

### 2.10. Analysis of Cell Apoptosis

Apoptosis was investigated using the AnnexinV Alexa Fluor647/PI kit (FMSAV647-100) by flow cytometry. LLC-PK1 cells were detached using Accutase Cell Detachment Solution (FMS-AT 100) for 5 min and then centrifuged at 2000 rpm for 5 min. The cells were washed twice with PBS and re-suspended with 500 μL of binding buffer at a concentration of 10^6^ cells/mL. Then, 5 μL of AF647-conjugated AnnexinV and 10 μL of PI were added to the cells, which was followed by incubation at room temperature for 15 min in the dark. Flow cytometry was carried out in a Becton Dickinson Accuri C6 FACSCalibur cytometer (BD Accuri C6 Plus, BD Medical Devices (Shanghai) Co., Ltd., Shanghai, China).

### 2.11. Statistical Analysis

The concentration of drugs that caused 80% cellular cytotoxicity is referred to as CC_80_. Experiments were performed in triplicate. Data are represented as the mean ± standard deviation (SD). Statistical differences were determined using a paired *t*-test and a one-way ANOVA using the Prism 8.0 software (GraphPad Software,). A *p*-value <0.05 was selected to indicate significance.

## 3. Results

**Figure 1:**
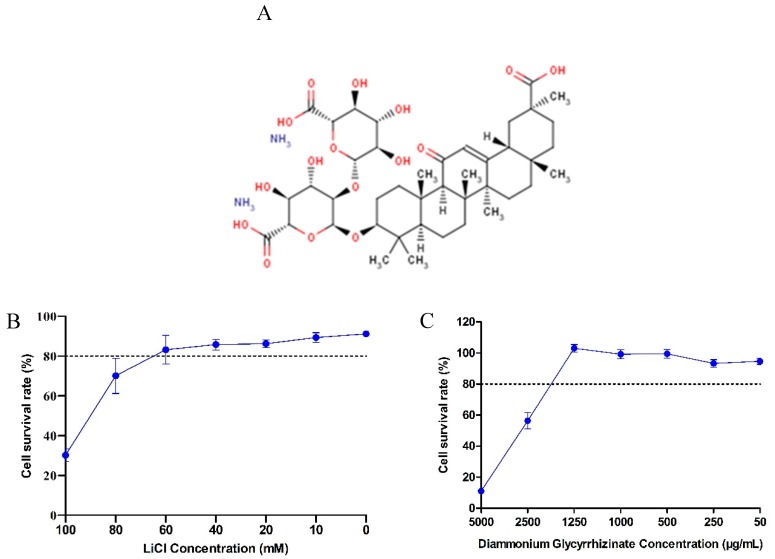
Cytotoxic effect of LiCl and DG treatment in LLC-PK1 cells. (**A**) Chemical structure of diammonium glycyrrhizinate (DG). Cells were treated with 0–100 mM LiCl (**B**) or 50–5000 μg/mL DG (**C**) for 36 h. The relative cell viability was evaluated by the CCK-8 Kit according to the manufacturer’s instructions. The data are expressed as the mean ± SD of three independent experiments. The dotted line indicates the 80% cytostatic concentration (CC_80_).

LiCl and DG cytotoxicity in LLC-PK1 cells. The cellular cytotoxic effect of LiCl and DG in LLC-PK1 cells was determined using the CCK8 kit. The structure of DG is shown in Figure 1A. The maximum LiCl and DG concentrations that resulted in a cell viability higher than 80% were 60 mM and 1250 μg/mL, respectively (Figure 1B,C). No differences in cell morphology compared to the mock-treated cells were observed (data not shown) at concentrations of 60 mM LiCl and 1250 μg/mL DG; therefore, 10–60 mM LiCl and 125–1250 μg/mL DG were used in subsequent antiviral assays. 

**Figure 2:**
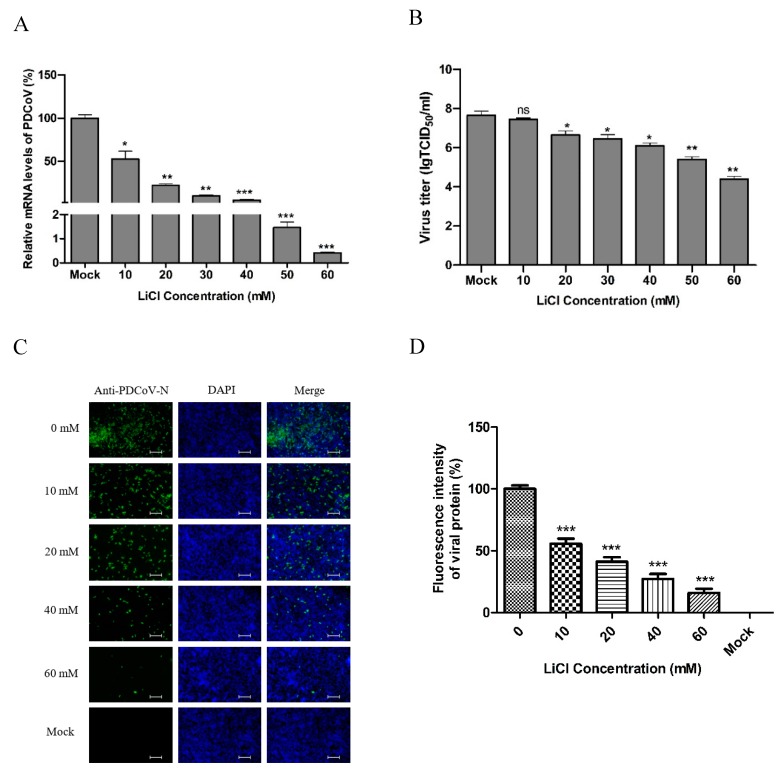
Antiviral effect of LiCl on porcine deltacoronavirus (PDCoV) replication. (**A**) The relative viral RNA level was determined by real-time qPCR. (**B**) The viral titer (log_10_ TCID_50_/_mL_) in LLC-PK1 cell lysates was calculated by the method of Reed and Muench. (**C**) At 24 h post-infection, PDCoV (MOI 0.05) replication in LLC-PK1 cells was determined by indirect immunofluorescence assay (IFA). Green fluorescence represents PDCoV replication, while blue fluorescence represents the nuclear distribution. (**D**) The fluorescence intensity in C was quantified with the software ImageJ. Quantification of PDCoV-infected cells from the IFA images is presented as percentage, taking 0 mM LiCl as 100%. Values represent the mean ± SD of three independent experiments; ns, not significant difference; * *p* < 0.05; ** *p* < 0.01; *** *p* < 0.001. Scale bar, 250 μm.

**Figure 3:**
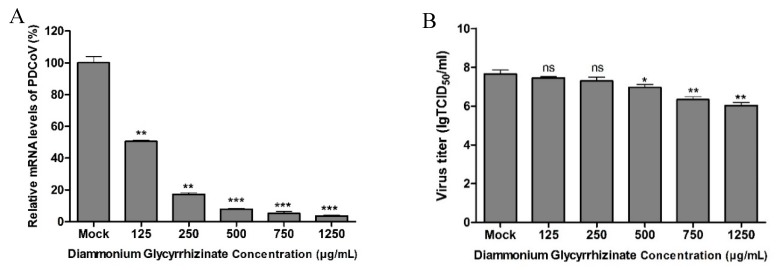

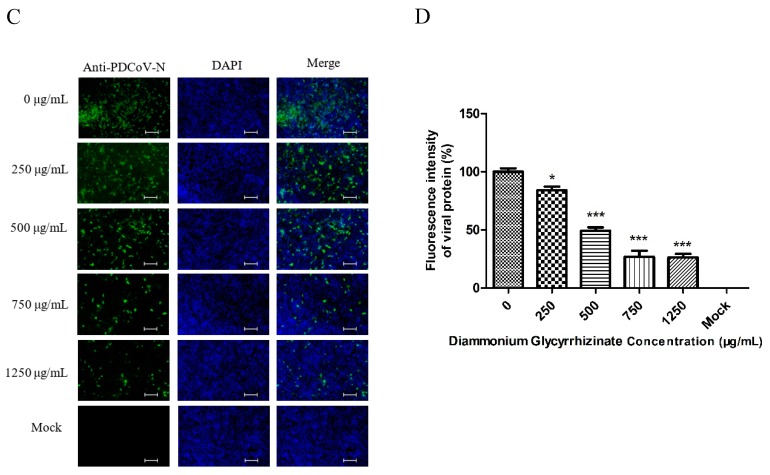
Antiviral effect of DG on PDCoV replication. (**A**) The relative viral RNA level was determined by real-time qPCR. (**B**). The viral titer (log_10_ TCID_50_/_mL_) in LLC-PK1 cell lysates was calculated by the method of Reed and Muench. (**C**) At 24 h post-infection, PDCoV (MOI 0.05) replication in LLC-PK1 cells was determined by IFA. Green fluorescence represents the PDCoV replication, while blue fluorescence represents the nuclear distribution. (**D**) The fluorescence intensity in C was quantified with the software ImageJ. Quantification of PDCoV infected cells from the IFA images is presented as percentage, taking 0 μg/mL DG as 100%. Values represent the mean ± SD of three independent experiments; ns, not significant difference; * *p* < 0.05; ** *p* < 0.01; *** *p* < 0.001. Scale bar, 250 μm.

LiCl and DG inhibit PDCoV replication. Next, we investigated the effect of LiCl and DG on the replication of PDCoV in LLC-PK1 cells. PK-1 cells were infected with PDCoV at MOI 0.05 for 1 h and treated with 10–60 mM LiCl or 125–1250 μg/mL DG for 24 h. The relative mRNA expression was detected by real-time qPCR. Treatment with 10–60 mM LiCl or 125–1250 μg/mL DG inhibited viral mRNA levels significantly (Figure 2A and Figure 3A). Infectious virus loads in cell culture supernatants were determined by TCID_50_. We found that 10–60 mM LiCl or 125–1250 μg/mL DG significantly inhibited viral replication compared to mock-treated cells (Figure 2B and Figure 3B). In IFA, the fluorescence intensity declined after treatment with 10–60 mM LiCl or 125–1250 μg/mL DG in a dose-dependent manner (Figure 2C and Figure 3C). The fluorescence intensity was quantified, showing that the number of infected cells decreased in a drug dose-dependent manner (Figure 2D and Figure 3D). These results indicate that LiCl and DG inhibit PDCoV replication in a dose-dependent manner.

**Figure 4:**
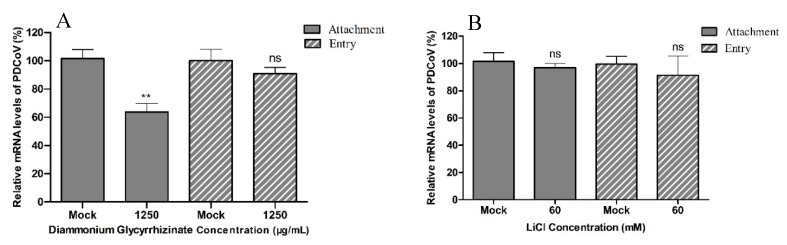
Effect of LiCl and DG on different stages of the PDCoV replication cycle. (**A**). The levels of vRNA in cells treated with 60 mM LiCl during viral attachment and entry were determined by real-time qPCR. (**B**) Same experiment as in A but using DG at 1250 μg/mL. Values represent the mean ± SD of three independent experiments; ns, no significant difference; * *p* < 0.05; ** *p* < 0.01; *** *p* < 0.001.

DG but not LiCl inhibits virus attachment to cells. To further explore in which step of the viral life cycle the drugs work, viral attachment and entry assays were performed in LLC-PK1 cells. There were no significant differences between mock-treated cells and cells treated with 60 mM LiCl in viral attachment or entry based on the mean relative viral mRNA levels (Figure 4A). In the entry experiments, there were no significant differences between mock-treated cells and cells treated with 1250 μg/mL DG in the mean relative viral mRNA levels. In viral attachment assays, DG treatment led to a significant reduction of viral mRNA levels compared to mock-treated cells (Figure 4B).

**Figure 5:**
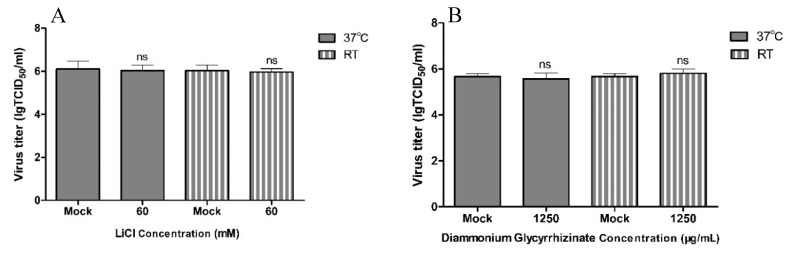
The viricidal effect of LiCl and DG on PDCoV. (**A**) PDCoV virions were incubated with 60 mM LiCl at 37 °C or room temperature. The viral titers (log_10_ TCID_50_/_mL_) were calculated by the method of Reed and Muench. (**B**) Same experiment as in A but using DG at 1250 μg/mL. Values represent the mean ± SD of three independent experiments, ns, no significant difference; * *p* < 0.05; ** *p* < 0.01; *** *p* < 0.001.

LiCl and DG have no viricidal effect on PDCoV virions. To understand whether LiCl and DG have a direct (viricidal) effect on virions, we incubated PDCoV (MOI 0.05) with 60 mM LiCl or 1250 μg/mL DG for 2 h at room temperature or 37 °C before infection of LLC-PK1 cells. The infectious titer was determined by TCID_50_ at 48 hours postinfection (hpi). We found that none of the drugs directly inhibited PDCoV infectivity (Figure 5A,B).

**Figure 6:**
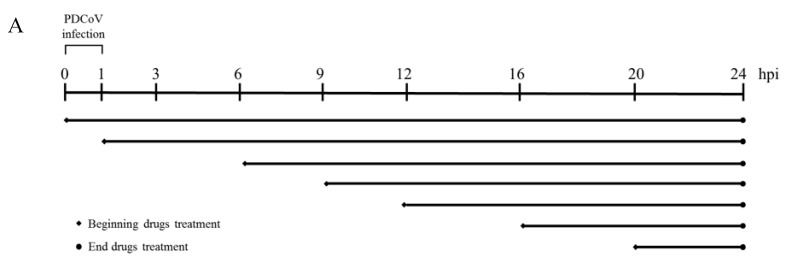

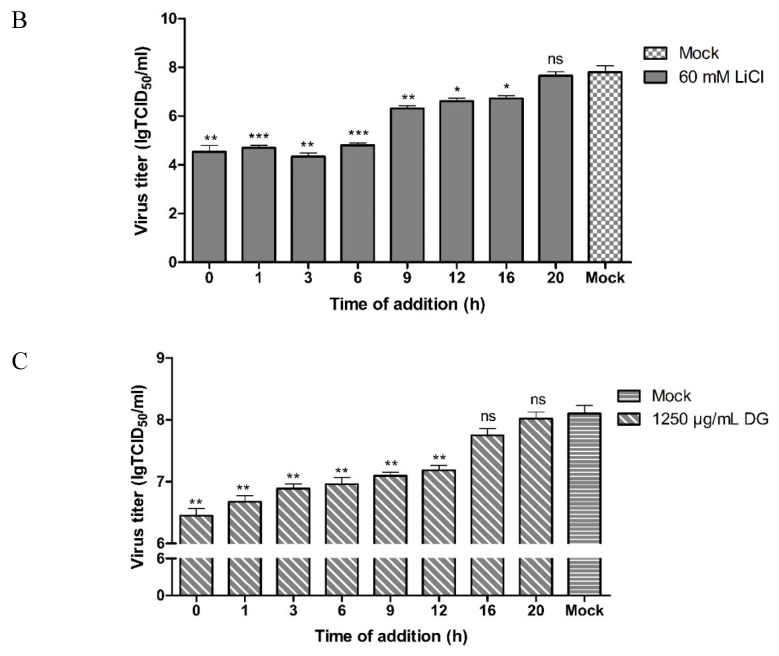
Time-dependent effect of LiCl and DG on PDCoV replication. (**A**) Outline of the experimental set up. (**B**,**C**) LLC-PK1 cells were incubated with PDCoV (MOI 0.05) for 1 h, followed by treatment with 60 mM LiCl (**B**) or 1250 μg/mL DG (**C**) at the indicated time (hpi). The viral titer in LLC-PK1 cell lysates was calculated using the method of Reed and Muench. Values represent the mean ± SD of three independent experiments; ns, no significant difference; * *p* < 0.05; ** *p* < 0.01; *** *p* < 0.001.

The antiviral effects of LiCl and DG occur at the early stage of PDCoV replication. To further illustrate the effect of LiCl and DG on PDCoV infection, we performed time of addition experiments (Figure 6A). Therefore, LiCl or DG was added to infected LLC-PK1 cells at different times. In the ‘0 hpi’ experiment, drugs and virus were added together to LLC-PK1 cells for 1 h. The cells were then washed, and the incubation was continued in the presence of the drugs. In the other experiments, LLC-PK1 cells were infected with PDCoV for 1 h, the cells were then washed, and the drugs were added at 1, 3, 6, 9, 12, 16, and 20 hpi. In all experiments, the cells were infected with PDCoV [ multiplicity of infection (MOI) = 0.05], and 60 mM LiCl or 1250 μg/mL DG was added. The viral contents were significantly reduced in cells treated with LiCl at 0 to 16 h, but no significant reduction was detected after 16 h (Figure 6B). For DG, the latest effective time point of drug treatment was 12 hpi (Figure 6C). Therefore, we can conclude that the antiviral effects of LiCl and DG occur at the early stages of PDCoV replication. 

**Figure 7:**
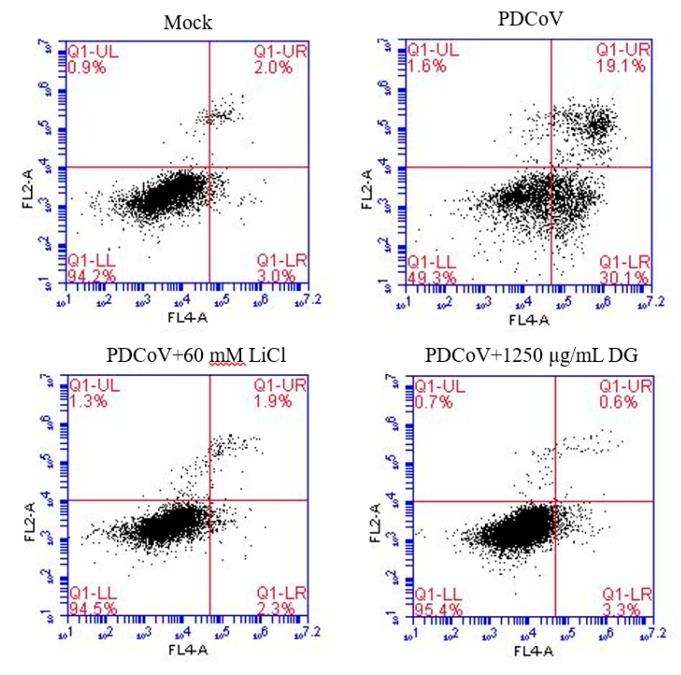
Effect of LiCl and DG on cell apoptosis caused by PDCoV infection. The cells were incubated with PDCoV (MOI 0.05) for 1 h. Thereafter, the supernatant was removed, and DMEM supplemented with 60 mM LiCl or 1250 μg/mL DG was added. At 36 hpi, the rates of cell apoptosis were analyzed by flow cytometry.

LiCl and DG inhibit LLC-PK1 apoptosis caused by PDCoV infection. The AnnexinV Alexa Fluor647/PI kit was used to detect cell apoptosis. We found that PDCoV could cause apoptosis within 36 hpi. The percentages of apoptotic cells infected by mock-treated, LiCl-treated viruses, and DG-treated viruses were 30.1%, 2.3%, 3.3%, respectively (Figure 7). Additionally, both drugs presented the same apoptosis-inhibition trend. Therefore, it can be concluded that LiCl and DG can inhibit PK1 cell apoptosis caused by PDCoV infection.

## 4. Discussion

PDCoV infection can cause vomiting and watery diarrhea in piglets and seriously damage the growth of the pig industry. Until now, there are no records of clinically effective agents for the treatment of PDCoV infection, and no compound has shown antiviral activity [35]. Therefore, there is an urgent need to develop a good approach for preventing PDCoV infection. Because neither specific antiviral drugs nor vaccines are available for newly emerging viruses and it is not possible to develop small-molecule drugs and vaccines that act on PDCoV in a short period of time, the novel use of traditional drugs has long been considered to be an important means of finding new therapies targeting new infectious diseases [36]. It has been reported that LiCl and DG, which are often deemed broad-spectrum antiviral reagents, have antiviral properties against coronaviruses such as SARS-CoV, IBV, and PEDV. However, it remains unknown whether LiCl or DG has an inhibitory effect on PDCoV infection.

In this study, we used real-time qPCR, TCID_50_, and IFA to analyze PDCoV infection. Real-time qPCR was used to analyze the level of PDCoV mRNA expression during PDCoV replication. The infectious titer was determined by TCID_50_, and the fluorescence signals detected by IFA were representative of the level of PDCoV protein expression. We found that LiCl and DG inhibited the replication of PDCoV in LLC-PK1 cells in a dose-dependent manner. By performing time-of-addition experiments, we aimed to identify the stage of the viral replication cycle targeted by the drugs. We found that both drugs can reduce viral RNA in cell lysates at the early stages of PDCoV replication. 

The viral replication cycle includes attachment, entry, transcription, replication, gene expression, assembly, maturation, and release. We found that DG can inhibit the attachment of the virus to the cell surface, which is consistent with the results of a recent report showing that glycyrrhizin achieved a dose-dependent inhibition of the replication of HIV-1 in MOLT-4 cells, and could interfere with virus–cell binding [37]. However, the specific mechanism needs further investigation. Future experiments will determine if DG can be used not only as a treatment but also for preventing PDCoV infection. Because DG has a good water solubility, it can be used as aerosol disinfectant in piggeries to prevent the outbreak of porcine epidemic diarrhea. According to the antiviral effect, the antiviral activity of LiCl is better than that of DG, and the toxicity of DG is less than that of LiCl. We investigated the effect of LiCl combined with DG on the replication of PDCoV in LLC-PK1 cells. There were no significant differences between LiCl-treated cells and cells treated with LiCl and DG in the mean relative viral mRNA levels, indicating that there is no synergy in the inhibition of viral replication when both drugs are used in combination (data not shown).

We also found that LiCl and DG could inhibit LLC-PK1 cell apoptosis caused by PDCoV infection similarly to the results of a previous report [28]. Apoptosis is considered a host innate defense mechanism to eliminate virus-infected cells. However, some viruses use and trigger apoptosis to facilitate the release of viral progeny for further dissemination. This can be fundamental for viral pathogenesis and disease development to promote cell death and tissue injury. Previous studies showed that PDCoV induces caspase-dependent apoptosis through the activation of the cytochrome c-mediated intrinsic mitochondrial pathway [38]. Whether LiCl and DG inhibit PDCoV-induced apoptosis through suppression of the cytochrome c-mediated intrinsic mitochondrial pathway needs to be further studied.

In conclusion, our results indicate that LiCl and DG have potential as effective anti-PDCoV drugs. During an outbreak of porcine epidemic diarrhea, LiCl and DG could be added to drinking water at the appropriate dose or be given as an intramuscular drug for treatment at the early onset of an epidemic. Further studies are required to explore their antiviral effect against other CoVs and their mechanism of action to control PDCoV infection in vivo.
